# Monitoring Circulating Tumor DNA in Untreated Non-Small-Cell Lung Cancer Patients

**DOI:** 10.3390/ijms23179527

**Published:** 2022-08-23

**Authors:** Woo Kyung Ryu, Sekyung Oh, Jun Hyeok Lim, Seung Jae Lee, Hyun-Tae Shin, Jeong-Seon Ryu

**Affiliations:** 1 Department of Internal Medicine, Inha University Hospital, Incheon 22332, Korea; 2 Department of Medical Sciences, Catholic Kwandong University College of Medicine, Incheon 22711, Korea; 3 DNA Link, Inc., Seoul 03721, Korea; 4 Department of Dermatology, Inha University Hospital, Incheon 22332, Korea; 5 Research Center for Controlling Intercellular Communication (RCIC), College of Medicine, Inha University, Incheon 22212, Korea

**Keywords:** non-small-cell lung cancer, circulating tumor DNA, monitoring

## Abstract

Circulating tumor DNA (ctDNA) has been utilized to monitor the clinical course of patients of non-small-cell lung cancer (NSCLC) who receive therapies targeting druggable mutations. However, despite providing valuable information on how NSCLC would naturally progress, the clinical utility of ctDNA for clinical-course monitoring and prediction of treatment-naïve NSCLC patients without druggable mutations remain unknown. We longitudinally followed a total of 12 treatment-naïve NSCLC patients, who did not harbor *EGFR* and *ALK* mutations, by collecting clinical information, radiological data, and plasma samples. Changes in ctDNA levels and tumor burden (TB) were compared with each other. New metastasis development, volume doubling time (VDT), and overall survival (OS) were analyzed regarding ctDNA detection at diagnosis. ctDNA was detected in the plasma of seven (58.3%) patients. Changes in ctDNA levels correlated with those in TB in a substantial fraction (57.1%) of patients and was also associated with brain metastasis, tumor necrosis, or pneumonia in other patients. All patients with ctDNA detection developed new metastasis during follow-ups in the organs that had been devoid of metastasis at diagnosis. The patients without ctDNA detection did not develop new metastasis (median duration of follow-ups: 9.8 months). In addition, patients with ctDNA detection had shorter VDT (*p* = 0.039) and worse OS (*p* = 0.019) than those without ctDNA detection. The natural course of NSCLC progression can be monitored by measuring ctDNA levels. Detection of ctDNA at diagnosis can predict development of new metastasis, rapid tumor growth and poor survival of NSCLC patients.

## 1. Introduction

Easily obtainable through liquid biopsy, circulating tumor DNA (ctDNA) has been of great interest to those who seek cancer biomarkers [1,2]. In non-small cell lung cancer (NSCLC), however, the clinical utility of ctDNA has been limited to detecting mutations in the genes that encode druggable proteins such as epidermal growth factor receptor (EGFR) and anaplastic lymphoma kinase (ALK). In addition, the current clinical-course monitoring of NSCLC by using ctDNA usually examines a single druggable driver gene in patients with targeted therapy [3].

While the majority of NSCLC patients receive anti-cancer treatments, a substantial proportion of patients (~20%) do not [4]. Elucidating the clinical course of such treatment-naïve patients, who currently are rarely followed up in most cases, would provide us with unprecedented, valuable information on how NSCLC naturally progresses in relation to patient survival [5]. Genetic markers as carried by ctDNA would help reveal molecular paths that NSCLC would naturally follow in the absence of therapy. Notably, the two most commonly adopted druggable mutations in monitoring NSCLC using ctDNA, those in *EGFR* and *ALK*, are recoverable in at most 20% of patients [6]. Whether the remainder of NSCLC patients without the druggable mutations would benefit from using ctDNA for clinical-course prediction or monitoring remains unclear.

In this vein, we investigated whether ctDNA obtained from treatment-naïve NSCLC patients could provide us with critical information on the natural clinical course when mutations affecting the druggable target genes are absent. We conducted longitudinal assessments of ctDNA levels, mutation maker fluctuations, and clinical features.

## 2. Results

### 2.1. Patient Characteristics and ctDNA Detection

**Figure 1:**
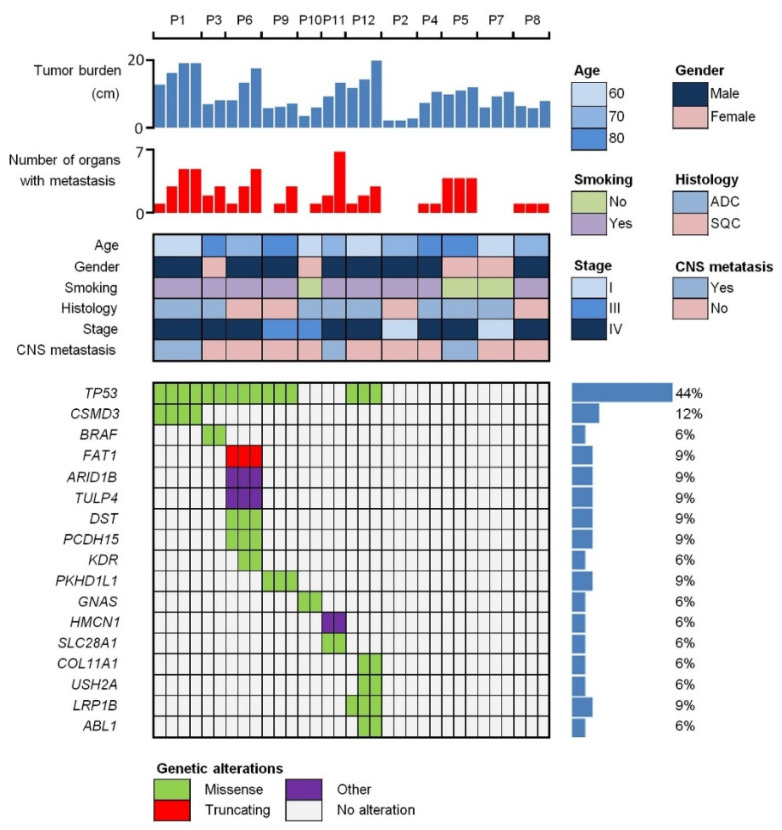
Somatic mutation profiles of circulating tumor DNA (ctDNA) and clinical characteristics. For each patient, the leftmost column represents T1 (baseline). In 7 patients (P1, P3, P6, P9, P10, P11, and P12), at least one mutation was detected in ctDNA, whereas no mutation was detected in 5 patients (P2, P4, P5, P7, and P8). Tumor burden showed a tendency to increase in all patients except P8.

Out of the 12 patients included in this study, eight (83.3%) were male and nine (75.0%) had smoking history (Figure 1). While eight (83.3%) patients had adenocarcinoma, eight (83.3%) were diagnosed with stage IV NSCLC. Metastatic lesions were newly developed in seven patients during follow-ups (Table 1). Pneumonia was observed in two patients (P8 and P12) during follow-ups. Necrosis of primary tumor was observed in two patients (P3 and P8). TB gradually increased in 11 (91.7%) patients except P8. In P8, the size of the solid tumor temporarily decreased slightly in proportion to the increase in necrotic area. The median duration of follow-ups was 9.8 months. Death was observed in 11 patients. The median survival of the patients was 7.7 months.

Several filtering steps were applied to sieve out putative germline and false variants (see Methods). A total of 66 somatic variants were identified (Table S1). The somatic variants were detected in the plasma of seven (58.3%) patients. The median ctDNA level was 9.1 hGE/mL (range: 0.8–108.5) and the median variant allele frequency was 4.2% (range: 0.1–21.9). Patients with detected ctDNA showed a trend to be male, have smoking history, have adenocarcinoma, and/or be diagnosed at advanced stages. The most common mutations detected were in *TP53* (five patients (41.7%)), followed by those in *CSMD3*, *FAT1*, *ARID1B*, *TULP4*, *DST*, *PCDH15*, *PKHD1L1*, and *LRP1B*. Variants, including both germline and somatic, with sufficient amounts of DNA were validated with ddPCR. The results of validation ddPCR were comparable to those of NGS in the patients with detected ctDNA (Table S2).

### 2.2. Change in ctDNA Levels and Tumor Burden

**Figure 2:**
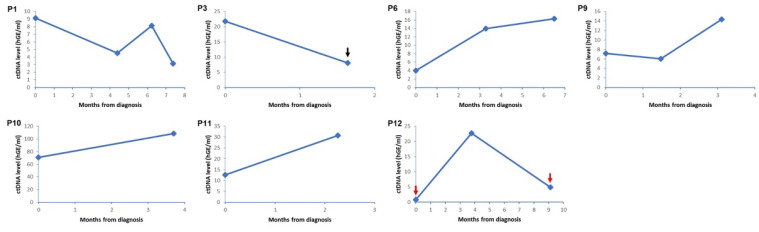
Changes in circulating tumor DNA (ctDNA) level of mutated gene in longitudinal plasma samples for patients with detected ctDNA. Each circle indicates a time point for plasma sampling and imaging test. In P3, the size of the primary tumor slightly increased whereas the area with tumor necrosis decreased at the second follow-up period (black arrow). In P12, pneumonia accompanied at diagnosis and the third follow-up period (red arrow).

Four patients (57.1%) with detected ctDNA (P6, P9, P10, and P11) displayed a tendency of increasing ctDNA levels over time, which may correspond to an increase in TB (Figure 2). By contrast, changes of ctDNA levels in the other three patients (P1, P3 and P12) showed discordance with an increase in TB. Several distinct clinical features accompanied these patients. In P1, although the primary tumor size gradually decreased, the total TB gradually increased as the size of the brain metastasis increased. In P3, the size of the primary tumor slightly increased whereas the area with accompanied tumor necrosis decreased. Pneumonia occurred at the diagnosis and the third follow-up period in P12.

### 2.3. Volume Doubling Time, Development of Metastasis and Overall Survival Regarding ctDNA Detection at Diagnosis

**Figure 3:**
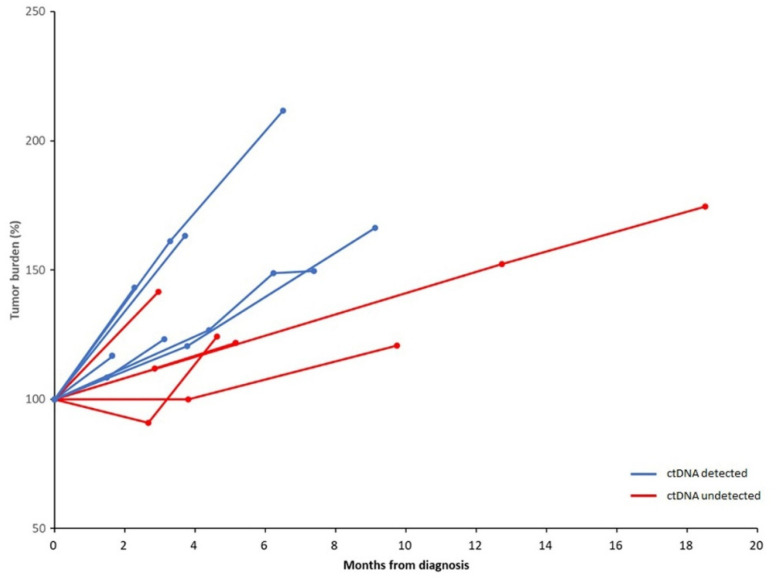
Changes in tumor burden in patients by detection of circulating tumor DNA (ctDNA). Patients with detected ctDNA showed rapid tumor growth when compared with those without detected ctDNA (median value of volume doubling time: 2.4 versus 5.9 months, *p* = 0.035).

**Figure 4:**
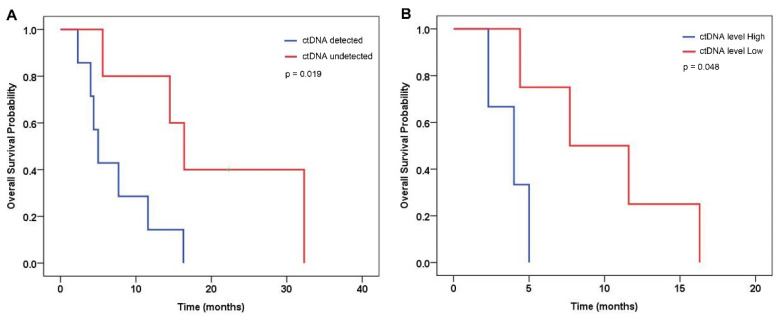
Kaplan–Meier curve for overall survival by (**A**) detection of circulating tumor DNA (ctDNA), and (**B**) ctDNA level. CtDNA levels were dichotomized by the median value.

The median value of VDT in the patients whose ctDNA was detected at diagnosis was 2.4 months. The patients whose ctDNA was not detected displayed a longer median VDT, 5.9 months (*p* = 0.039) (Figure 3). In all the patients with detected ctDNA, new metastasis arose during follow-ups in the organs that had not had metastasis at the time of diagnosis (Table 1). The patients lacking detectable ctDNA, however, did not show metastasis in a new organ. Of note, the patients with detected ctDNA had a significantly worse OS than those without detected ctDNA (a median OS of 5.0 months versus 16.4 months, log-rank *p* = 0.019) (Figure 4). Moreover, patients with high-level ctDNA had worse OS compared with those with low-level ctDNA (a median OS of 4.0 months versus 7.7 months, log-rank *p* = 0.048).

## 3. Discussion

We have shown here that changes in ctDNA levels correlated with TB in a substantial fraction of treatment-naïve NSCLC patients without the druggable mutations, but it could be affected by various clinical factors. We also noted that the natural course of NSCLC progression could be predicted by detecting ctDNA. We found that patients with ctDNA detected at diagnosis had shorter VDT, new metastases and worse OS during follow-ups.

The ctDNA levels of patients as measured in hGE in this study were lower than those of patients receiving chemotherapy in other studies [7,8]. This indicates that a fraction of treatment-naïve NSCLC patients could already harbor a considerable amount of ctDNA, which could be further increased by treatment-induced cancer cell death. Previous studies have suggested that changes in ctDNA levels can be positively correlated with TB, of the patients who receive chemotherapy [7,9]. Our results showed that alteration in ctDNA levels correlates with TB in some treatment-naïve patients, whereas it does not in others. We speculate that the relatively low amount of ctDNA detected in the treatment-naïve patients could easily be affected by clinical factors. Increased size of brain metastasis may account for the increased overall TB while the primary tumor size shrank (P1). As the blood–brain barrier could prevent ctDNA from entering the circulation, the increased size of brain metastasis may not be reflected in ctDNA [10]. The fall in the ctDNA level could be also explained by alteration of the area with tumor necrosis (P3) [3]. In addition, we observed decreased ctDNA levels when pneumonia accompanied, in one patient (P12). In this patient, there was no specific finding that could affect the ctDNA level except for pneumonia. We speculate that the ctDNA concentration in plasma was relatively decreased by the increased cell-free DNA due to the accompanying pneumonia [11]. 

Our results showed that the patients with ctDNA detection would develop accelerated tumor growth and new metastasis. The short VDT of the patients with ctDNA detection could reflect their high tumor proliferation rate [12]. Notably, ctDNA detection was strongly associated with new metastasis. We speculate that a substantial proportion of ctDNA detected in the treatment-naïve NSCLC patients could have come from a subset of metastasizing cancer cells, which were dying by inadequately engaging the anoikis resistance [13]. The high-level tumor proliferation and new metastasis observed in patients with ctDNA detection may account for the poor prognosis of these patients.

Patients with *EGFR-* or *ALK*-positive NSCLC were excluded to prevent inclusion of a significant proportion of patients with specific druggable driver mutations that could affect prediction of prognosis or monitoring of natural course. Nevertheless, one of the currently druggable mutations, *BRAF V600E*, was found in one patient (P3). However, unlike *EGFR* or *ALK*, *BRAF* has relatively low prevalence in lung cancer, and this study was conducted with untreated patients. Therefore, we suggest that the inclusion of one patient with *BRAF V600E* would not significantly affect the study outcome. Although *KRAS* mutation is one of the significant mutations in lung cancer, *KRAS* mutation was not detected in the plasma of any patient in this study. It has been reported that the prevalence of *KRAS* mutation in lung cancer is significantly lower in Asian populations than in Western populations [14,15]. We suggest that the absence of detection in *KRAS* mutations in Korean patients of this study reflects the low prevalence of *KRAS* mutations in Asians.

Our results should be interpreted with caution. First, this study included a relatively small number of samples. However, finding a sizable number of appropriate treatment-naïve patients, who also received follow-ups with imaging studies and ctDNA isolation, requires a huge patient population. In our cohort, only 12 patients (0.3%) met all the criteria among 3,740 patients diagnosed as NSCLC. Second, somatic mutations recovered from the ctDNA were not compared with those from the corresponding tumor specimens. However, we undertook every effort to filter out false signals in this study. Third, this study did not show whether ctDNA levels were useful as a prognostic factor compared with clinical stage or treatment. Therefore, prospective studies showing that the application of ctDNA levels have the benefit of earlier or more specific clinical interventions will be needed. Additionally, it is not clear whether it is clinically useful for untreated patients to know that they would be progressive. Sometimes this can seriously impair the quality of life.

To summarize, this study reveals that natural course could be monitored using ctDNA levels in NSCLC patients, but various clinical factors such as brain metastasis, tumor necrosis or infection can affect the ctDNA levels. Our results also imply that detection of ctDNA at diagnosis can predict rapid tumor growth, development of new metastasis and poor survival of the NSCLC patients. This study indicates that natural course could be monitored or predicted with individually harbored somatic mutations in ctDNA of NSCLC patients.

## 4. Materials and Methods

### 4.1. Patients and Radiological Evaluation

**Figure 5:**
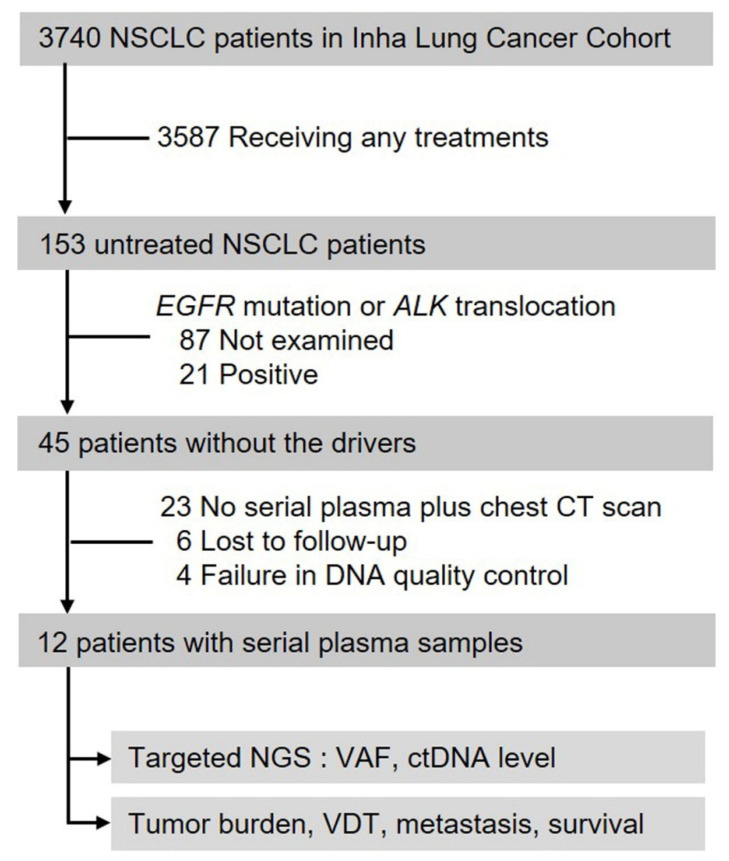
Flowchart of patient selection. NSCLC, non-small cell lung cancer; EGFR, epidermal growth factor receptor; ALK, anaplastic lymphoma kinase; CT, computed tomography; NGS, next-generation sequencing; VAF, variant allele frequency; hGE, haploid genome equivalent; VDT, volume doubling time.

This study was approved by the Institutional Review Board of Inha University Hospital (2005-03-001-015) and informed consents were obtained from patients. A total of 3740 patients who had been histologically diagnosed as NSCLC between January 2008 and March 2017 were identified in the Inha Lung Cancer Cohort (Inha University Hospital, Incheon, South Korea) and initially considered for this study (Figure 5). Among them, 153 patients did not take any anti-cancer treatment. The treatment refusal was based on (1) either severe comorbid diseases or more than three markings in the Eastern Cooperative Oncology Group performance status (n = 87); (2) patients’ wish not to take treatment (n = 39); (3) poor economic status (n = 18); and (4) unknown reasons (n = 9). Finally, a total of 12 patients (P1 to P12) were included in this study. All clinical information regarding age, gender, smoking history, histology, stage, and site of metastasis, was prospectively acquired.

Radiological evaluations were performed at the time of diagnosis, and the ensuing follow-ups occurred at an average of three-month intervals (range: 2–5). The stage was estimated using the 8th edition of the Tumor, Node, Metastasis classification [16]. Tumor burden (TB) was measured by the sum of greatest diameters of target lesions with the standard of RECIST v1.1 [17]. All methods were performed in accordance with the relevant guidelines and regulations.

### 4.2. Plasma Samples and DNA Extraction

A total of 37 serial plasma samples of the patients included in the study were drawn at the same time as the radiological evaluation and subsequently stored at −80 °C until use. Cell-free DNA was extracted from each plasma sample using a QIAamp Circulating Nucleic Acid Kit (QIAGEN, Hilden, Germany) following the manufacturer’s instructions and eluted with 30 μL of Buffer AVE. Extracted DNA amounts were measured with Qubit 2.0 (Life Technologies, Grand Island, NY, USA) and quality evaluated with Agilent 2100 bioanalyzer (Agilent, Santa Clara, CA, USA). Genomic DNA from frozen cell pellets was isolated using a QIAamp DNA Blood Mini Kit (QIAGEN, Hilden, Germany) following the manufacturer’s instructions, and eluted with 50 μL of Buffer EB. A QuickGene DNA Whole Blood Kit S (Kurabo, Osaka, Japan) was used for the buffy coat sample, while a QuickGene DNA Whole Tissue Kit S (Kurabo, Osaka, Japan) was used for the tissue sample. All extraction steps followed the manufacturer’s protocols. DNA amounts and purity were measured with nanodrop 8000, Qubit 2.0, and were quality evaluated using 1.0% agarose gel electrophoresis.

### 4.3. The Custom Lung Cancer Panel for Korean Population

We developed a custom high-depth sequencing panel for the detailed profiling of Korean lung cancer. Results of somatic mutations from the lung cohort of The Cancer Genome Atlas (TCGA) (https://gdac.broadinstitute.org, accessed on 20 October 2021) and research on the Korean population [14,18,19] were merged to generate the ethnic-specific custom panel. Then, using the Agilent SureSelect custom design platform, we designed the panel with 113 genes with a sample frequency of more than 5% in the aforementioned studies (Table S3). It had a 1.705 Mbp region size and covered all axons of the target genes.

### 4.4. Targeted Sequencing

Target enrichment bait was designed using Agilent SureDesign software. Target regions from 200 ng or 1 μg of genomic DNA were captured using an Agilent SureSelectXT Custom kit with target enrichment bait, following the manufacturer’s protocols (Agilent, Santa Clara, CA, USA). Briefly, DNA was sheared by the Covaris system (Covaris, Woburn, MA, USA) and purified using Agencourt AMPure XP beads (Beckman Coulter, Brea, CA, USA). The fragment ends were repaired and adaptors were ligated to the fragments. The resulting DNA library was purified using Agencourt AMPure XP beads and amplified by PCR. The quality and quantity of the DNA library was assessed with an Agilent 2100 Bioanalyzer. The DNA library was captured by hybridization to the biotinylated RNA library baits. Bound genomic DNA was purified with streptavidin-coated magnetic Dynabeads (Invitrogen, Carlsbad, CA, USA) and then re-amplified. The targeted DNA library was sequenced on Illumina Hiseq2500 with 100 base-pair paired-end reads using recommended protocols from the manufacturer (Illumina, San Diego, CA, USA). In this study, samples were sequenced to an average of 10,000× on raw data.

### 4.5. Preprocessing and Variant Analysis

Sequence QC was performed through FastqQC 0.11.2 (Andrews, S. (2010). FastQC. A quality control tool for high throughput sequence data. http://www.bioinformatics.bbsrc.ac.uk/projects/fastqc/, accessed on 20 October 2021), and was mapped to the human reference genome sequence NCBI b37 using bwa 0.7.12 [20]. BAM files were sorted and deduplicated with Picard Tools v2.2.1, and realigned with Genome Analysis Toolkit 3.5 (GATK) IndelRealigner, and base quality scores were recalibrated by the GATK base quality recalibration tool [21]. Variants were detected with LoFreq v2.1.3.1 with a default parameter setting [22]. Then, the variants’ additional information was annotated using ANNOVAR [23]. We applied several filtering steps to filter these putative germline and false variants: (i) variants with very high variant allele frequency (VAF) (≥97%), except for hotspot mutations; (ii) variants with high mean VAF (≥35%) at serial points, except for hotspot mutations; (iii) variants with population allele frequency >1% in normal samples in the population database; and (iv) other frequently detected variants that are likely to be alignment artifacts or are in hard-to-sequence regions, as curated by manual review using Integrative Genomics Viewer (IGV) [24]. Among the genetic mutations that passed through the filters, only those consistently found in more than two visits were used for analytical monitoring. If a variant was consistently detected in two-time points and not detected in some time point, we checked and filled up the variants at the genomic position using IGV.

The circulating tumor DNA (ctDNA) levels were expressed in haploid genome equivalents per milliliter of plasma (hGE/mL) and calculated by multiplying the VAF of clonal mutations by the concentration of cfDNA (pg/mL of plasma) and dividing by 3.3, as previously described in the publication by Scherer et al. [25].

### 4.6. Digital Droplet PCR

The quality of cfDNA was verified by capillary electrophoresis (2100 Bioanalyzer, Agilent, Santa Clara, CA, USA) and fluorescence-based quantitation (Qubit, Invitrogen, Carlsbad, CA, USA). The QX200 Droplet Digital PCR (ddPCR) System provides absolute quantification of target DNA or RNA molecules for EvaGreen or probe-based digital PCR applications. ddPCR was performed using the QX200 Droplet Digital PCR System (Bio-Rad, Hercules, CA, USA) as per the manufacturer’s protocol with modification, as described below. The ddPCR mixture contained 10 μL of 2× ddPCR Supermix for probes (Bio-Rad, Hercules, CA, USA) (no dUTP), 0.5 μL 40× probe (10 µM), and 9.5 μL of the 10 ng cfDNA template. Every ddPCR run included negative template controls (NTCs) and positive template controls (PTCs) run. An amount of 20 μL of each well-mixed ddPCR mixture was transferred to a droplet generator cartridge (Bio-Rad, Hercules, CA, USA). After 70 μL of droplet generation oil (Bio-Rad, Hercules, CA, USA) was added into the oil wells, the cartridge was covered with a gasket and loaded onto a QX200 droplet generator (Bio-Rad, Hercules, CA, USA). The emulsions of droplets generated were transferred to a 96-well semi-skirted PCR plate (Eppendorf, Hamburg, Germany), sealed with PX1 PCR plate sealer (Bio-Rad), and subjected to amplification in a GeneAmp PCR System 9700 (Applied Biosystems, Waltham, MA, USA) (95 °C 10 min, 40 cycle (94 °C, 30 s; 55 °C, 1 min; ramp rate 2 °C/s); 98 °C 10 °C/min, 4 °C infinite). QuantaSoft™ software (version 1.7.4) was used to assign positive/negative droplets and convert counts to a copies/μL or fractional abundance value (% allelic frequency).

### 4.7. Statistical Analysis

CtDNA levels were analyzed as continuous or dichotomized variables based on the median value. Volume doubling time (VDT) was calculated by using an equation based on the modified Schwartz formula [26]. Overall survival (OS) was defined from the time of diagnosis until death as a result of all causes. If patients were still alive on the last follow-up date, they were censored on that day. Patients were classified into two groups with the presence of detected ctDNA. Changes in ctDNA levels were compared with those of TB. VDT was compared between groups using t-test. The median OS was estimated by the Kaplan–Meier method and compared using log-rank test. To estimate statistical significance, two-tailed hypothetical testing was performed with rejection of the null hypothesis when *p* < 0.05. All statistical analyses were performed using the IBM SPSS statistical software package (version 19.0; SPSS Inc., Chicago, IL, USA).

## Figures and Tables

**Table 1:** Patient characteristics and circulating tumor DNA detection.

Patient	Time ^a^	ctDNA Detection	VDT (Months)	Site of Metastasis	Pneumonia	Tumor Necrosis
P1	T1	Yes	4.3	Br		
	T2			Br, Lu, Pl		
	T3			Br, Lu, Pl, Bo, Al		
	T4			Br, Lu, Pl, Bo, Al		
P2	T1	No	7.2	None		
	T2			None		
	T3			None		
P3	T1	Yes	2.4	Bo, Pl		Yes
	T2			Bo, Pl, Cw		Yes
P4	T1	No	2.0	Pl		
	T2			Pl		
P5	T1	No	5.9	Bo, Br, Lu, Pl		
	T2			Bo, Br, Lu, Pl		
	T3			Bo, Br, Lu, Pl		
P6	T1	Yes	1.6	Li, Bo		
	T2			Li, Bo, Pl		
	T3			Li, Bo, Pl, Pc, Al		
P7	T1	No	7.0	None		
	T2			None		
	T3			None		
P8	T1	No	4.9	Pl	Yes	Yes
	T2			Pl		Yes
	T3			Pl	Yes	Yes
P9	T1	Yes	4.3	None		
	T2			Li		
	T3			Li, Kd, Pt		
P10	T1	Yes	1.7	None		
	T2			Pl		
P11	T1	Yes	1.4	Br, Lu		
	T2			Br, Lu, Ad, Sp, Li, Pt, Aw		
P12	T1	Yes	4.7	Bo	Yes	
	T2			Bo, Lu		
	T3			Bo, Lu, Li	Yes	

^a^ T1 to T4 denote a time point for plasma sampling and imaging tests; ctDNA, circulating tumor DNA; VDT, volume doubling time; Br, brain; Lu, lung; Pl, pleura; Bo, bone; Al, abdominal lymph node; Cw, chest wall; Li, liver; Pc, pericardium; Kd, kidney; Pt, peritoneum; Sp, spleen; Aw, abdominal wall.

## Data Availability

The data presented in this study are available in the manuscript. Additional raw data are available on request from the corresponding author.

## Supplementary Materials

The following supporting information can be downloaded at: https://www.mdpi.com/article/10.3390/ijms23179527/s1.
